# Osteopontin Affects Insulin Vesicle Localization and Ca^2+^ Homeostasis in Pancreatic Beta Cells from Female Mice

**DOI:** 10.1371/journal.pone.0170498

**Published:** 2017-01-20

**Authors:** Anna Wendt, Inês G. Mollet, Anki Knutsson, Victor S. Bolmgren, Anna Hultgårdh-Nilsson, Maria F. Gomez, Lena Eliasson

**Affiliations:** 1 Unit of Islet Cell Exocytosis, Lund University Diabetes Centre, Department of Clinical Sciences in Malmö, Lund University, Clinical Research Centre, Malmö, Sweden; 2 Vessel Wall Biology, Department of Experimental Medical Science, Lund University, Biomedical Center, Lund, Sweden; 3 Unit of Vascular excitation-transcription (ET) coupling, Lund University Diabetes Centre, Department of Clinical Sciences in Malmö, Lund University, Clinical Research Centre, Malmö, Sweden; University of Bremen, GERMANY

## Abstract

Type 2 diabetic patients suffer from insulin resistance and reduced insulin secretion. Osteopontin (OPN), a versatile protein expressed in several tissues throughout the body including the islets of Langerhans, has previously been implicated in the development of insulin resistance. Here we have investigated the role of OPN in insulin secretion using an OPN knock out mouse model (OPN^-/-^). Ultra-structural analyzes of islets from OPN^-/-^ and WT mice indicated weaker cell-cell connections between the islet cells in the OPN^-/-^ mouse compared to WT. Analysis of the insulin granule distribution in the beta cells showed that although OPN^-/-^ and WT beta cells have the same number of insulin granules OPN^-/-^ beta cells have significantly fewer docked granules. Both OPN^-/-^ and WT islets displayed synchronized Ca^2+^ oscillations indicative of an intact beta cell communication. OPN^-/-^ islets displayed higher intracellular Ca^2+^ concentrations when stimulated with 16.7 mM glucose than WT islets and the initial dip upon elevated glucose concentrations (which is associated with Ca^2+^ uptake into ER) was significantly lower in these islets. Glucose-induced insulin secretion was similar in OPN^-/-^ and WT islets. Likewise, non-fasted blood glucose levels were the same in both groups. In summary, deletion of OPN results in several minor beta-cell defects that can be compensated for in a healthy system.

## Introduction

Osteopontin (OPN) was originally discovered in bone [1], but has since then been detected in various tissues including the islets of Langerhans [2]. OPN is mainly known as a secreted extracellular matrix protein although an intracellular version of the protein also exists. It is believed that extracellular and intracellular OPN serves different biological roles [3]. In rodents, all major islet cells including the beta cells express OPN [4, 5]. The role of OPN in islets is not fully established but OPN seems to prevent apoptosis and stimulate proliferation of islets and insulin producing cells, suggesting an overall islet protective role [4, 6, 7]. However, Serum OPN has been reported to be predictive of cardiovascular disease in patients suffering from type 1 diabetes [8] and in a recent study by Barchetta *et*.*al*., type 1 diabetic patients displayed higher serum OPN levels than the control group [9]. Interestingly, in the latter study high OPN levels correlated with a negative metabolic profile including higher blood pressure and body mass index, and lower HDL levels.

In the context of type 2 diabetes OPN has been suggested to be involved in adipose tissue inflammation and insulin resistance [10, 11]. Type 2 diabetes is characterized by both insulin resistance and impaired insulin secretion. Little is known about the effects of OPN on insulin secretion. Extracellular addition of OPN has a beta cell protective role against IL-1β induced cytotoxicity and helps normalizing glucose-induced insulin secretion in IL-1β treated cells [6]. Similarly, extracellular addition of OPN to islets from mildly diabetic rats was shown to improve their glucose-stimulated insulin secretion [4]. Both of these effects were suggested to, at least in part, act through regulation of NO production in the beta cells. Reports on direct effects of OPN on the insulin secretory machinery are scarce. Insulin is the major glucose lowering hormone in the body and as such its release is tightly regulated by a plethora of control mechanisms. The main trigger for insulin release is glucose. Increased blood glucose levels trigger the Ca^2+^ dependent release of insulin through a mechanism commonly referred to as the stimulus-secretion coupling pathway of the beta cells [12]. According to this well established theory glucose equilibrates across the beta cell membrane via low affinity glucose transporters. Inside the beta cell glucose is metabolized and the resulting increase in ATP at the expense of ADP triggers the closure of K_ATP_ channels in the cell membrane. This results in a depolarization of the cell membrane which ultimately leads to the opening of voltage-gated Ca^2+^ channels. The resulting increase in Ca^2+^ is the signal for insulin-containing vesicles to fuse with the membrane and release their content (reviewed in [13]). Before the insulin-containing vesicles can fuse with the cell membrane and release their cargo they have to be brought close to the cell membrane and rendered release competent. This is done through a series of events collectively called docking and priming [14, 15].

Here we have characterized islets of Langerhans from OPN^-/-^ mice with respect to morphology, Ca^2+^ handling, and capacity to secrete insulin.

## Materials and Methods

All chemicals were purchased from Sigma Aldrich (MO, USA) unless otherwise stated.

### Islet isolation and culture

All animal procedures were approved by the local ethics committee for use of laboratory animals in Malmö, Sweden (Malmö/Lunds djurförsöksetiska nämnd; approval number M105-15). The mice were sacrificed by cervical dislocation prior to experiments and all efforts were made to minimize any kind suffering. The generation of the OPN^-/-^ mouse has previously been described by Franzén *et*. *al*. [16]. Briefly, OPN deficient mice were generated through deletion of the promoter and exon 1–3 of the OPN gene. All mice were backcrossed to C57BL/6 several times. Homozygotic breeding of WT and OPN^-/-^ animals from these litters were then used for experiments.

Pancreatic islets from 12 week old female OPN^-/-^ and WT control mice were prepared by collagenase digestion as previously described [17]. The isolated islets were used for experiments immediately after isolation except for the Ca^2+^ imaging experiments. For these experiments the islets were cultured overnight in RPMI 1640 with 10 mM glucose, 10% FCS, 100 U/ml penicillin, and 100 μg/ml streptomycin at 37°C in a humidified cell incubator containing 5% CO_2_ and 95% air previous to the experiments.

### Histology

Pancreases from 12 week old female OPN^-/-^ [16] and WT control mice were dissected out after perfusion with Histochoice (Amresco). The pancreases were fixed in Histochoice for 48 h, embedded in paraffin and sectioned at 5 μm.

**Fig 1:**
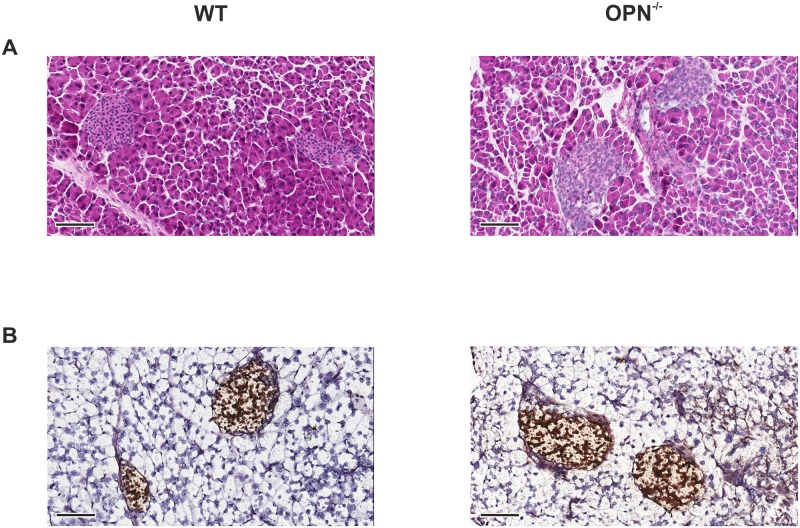
Pancreatic slices from OPN^-/-^ and WT mice have similar histology. Representative images from (A) eosin and hematoxylin stainings and (B) insulin stainings on pancreatic slices from WT (left) and OPN^-/-^ (right) mice. Scale bar 100 μm.

In the eosin and hematoxylin stainings (Fig 1A) the sections were deparaffinized and hydrated to water where after they were stained for 1 min in Harris Hematoxylin (Sigma; HHS32-1L). The slides were then rinsed in dH_2_O and quickly dipped in 0.5% acid alcohol before rinsing in dH_2_O again. The slides were then incubated in Scott's tap water (2 g/l sodium bicarbonate; 10 g/l anhydrous MgSO_4_) for 1 min and then quickly rinsed in dH_2_O. The slides were then incubated in 80% ethanol for 1 min and then submerged in eosin (Sigma; HT11032) for 20 seconds. The slides were finally dehydrated, cleared in xylene and mounted using pertex (Histolab; 00811). Slides from 13 animals per condition were prepared.

The ImmPRESS HRP Anti-Rabbit IgG (Peroxidase) Polymer Detection Kit (made in horse) was used for the insulin staining (Fig 1B) according to the manufacturer’s instructions. In short the sections were deparaffinized and hydrated to water. Endogenous peroxidase activity was quenched using 3% H_2_O_2_ in dH_2_O for 10 min. After rinsing, the sections were blocked in ready-made 2.5% horse serum for 20 min. The primary insulin antibody (AbCam; ab6382) was added at a concentration of 10 μg/ml diluted in 2.5% goat serum and incubated at 4°C overnight. After rinsing, the ready-made secondary antibody was added to the section and incubated for 30 min at room temperature. After rinsing, the sections were then incubated with the peroxidase substrate solution ImmPRESS DAB (Vector Laboratories; SK-4105) until the desired intensity developed. The sections were counterstained using Harris Hematoxylin (Sigma; HHS32-1L) for 5 min. After rinsing in tap water for 5 min the sections were dehydrated, cleared in xylene and mounted using pertex (Histolab; 00811). Sections from 13 animals per condition were prepared.

### Electron microscopy

**Fig 2:**
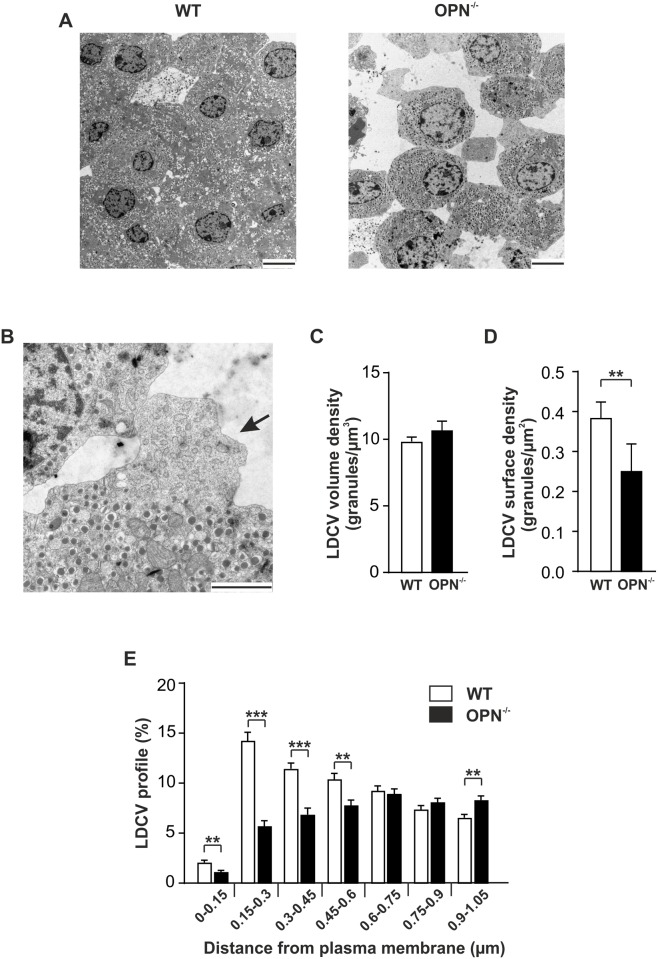
Altered cell-cell connections and insulin granule distribution in beta cells from OPN^-/-^ mice. (A) Ultrastructural images from a section of a WT (left) and OPN^-/-^ (right) islet. Scale bar 5 μM. (B) Ultrastructural images from a section of an OPN^-/-^ islet. The atypical structure on the beta cell is marked with a black arrow. Scale bar 2μM. (C, D, and E) Histogram of the calculated volume density (C), surface density (D), and distribution of the insulin containing large dense-core vesicles (LDCVs; E) in pancreatic beta cells from WT and OPN^-/-^ mice. Data are given as mean ± SEM from 24–26 cells taken from 3 animals per condition. ** p≤ 0.01; *** p≤ 0.001.

For transmission electron microscopy analyses (Fig 2), groups of 50 islets were initially fixed in 2.5% glutaraldehyde in Millonig’s buffer (2.26% NaH2PO4 and 2.52% NaON) for 2 h at 4°C and then post-fixed in 1.0% osmium tetroxide for 1h followed by dehydration and embedding in AGAR 100 (Oxfors Instruments Nordiska AB, Sweden). Embedded islets were cut into 70–90 nm thick sections, mounted onto Cu grids and contrasted with uranyl acetate and lead citrate before being examined with a JEM 1230 electron microscope (JEOL-USA, Inc., MA, USA). Granule diameters were measured using ImageJ (NIH, freeware) and estimated 3D diameter of the granules was calculated as described [18]. Volume granule density (Nv) and surface density (Ns) were calculated using an in-house Matlab program on MatLab7 (MathWorks, Natick, MA, USA) and methods described elsewhere [19]. Data are derived from 24–26 cells taken from 3 animals per condition.

### Ca^2+^ imaging

**Fig 3:**
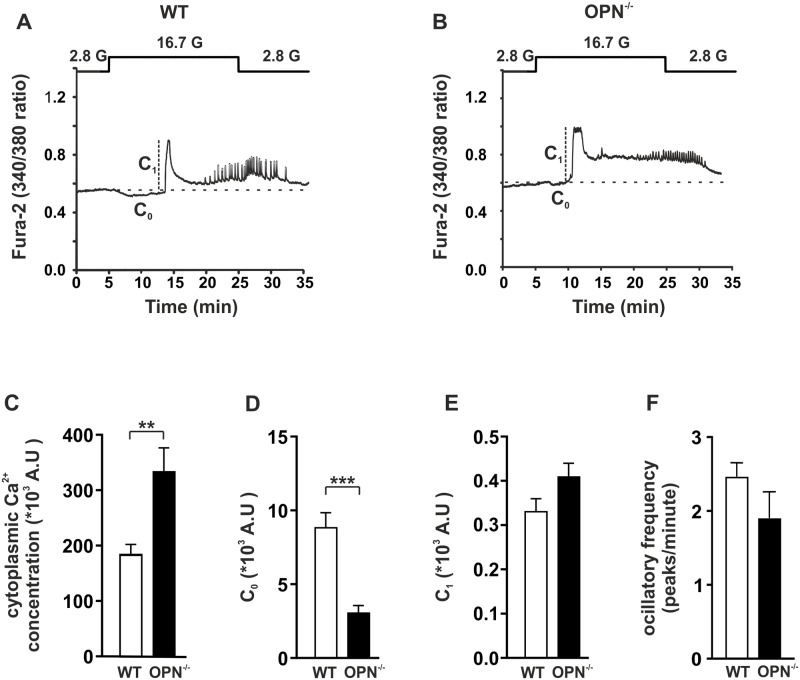
Example traces from intracellular Ca^2+^ measurements in WT and OPN^-/-^ islets. Representative examples of Ca^2+^ traces obtained from an islet from a WT (A) or OPN^-/-^ (B) mouse. Marked on the traces are the measurements mentioned in the text. C_0_ is the area of the Ca^2+^ dip that initially occurs upon glucose-stimulation. C_1_ is the amplitude of the first peak. 16.7 G is 16.7 mM glucose and 2.8 G is 2.8 mM glucose. The baseline is marked with a dashed line. Histogram of the cytoplasmic Ca^2+^ concentration (measured as area under the curve but above the baseline; C), C_0_ (D), C_1_ (E), and the frequency of ocillations during the sustained phase (F) in islets from WT (white bars) and OPN^-/-^ (black bars) mice. Data are obtained from 16–21 islets from 3 mice per condition. ** p≤ 0.01; *** p≤ 0.001.

Mouse islets were isolated and incubated in medium overnight as described above. At the onset of the experiment the islets were loaded with 4 μM Fura-2-acetoxymethyl ester (TefLabs, Austin, TX, USA) for 40 min, followed by a 30 min de-esterification in imaging buffer. The imaging buffer contained (in mM) 3.6 KCl, 0.5 MgSO_4_, 2.5 CaCl_2_, 140 NaCl, 2 NaHCO_3_, 0.5 NaH_2_PO_3_ 5 HEPES and 2.8 or 16.7 glucose as indicated in the figures; pH of the buffer was set to 7.4. A buffer with high concentration of K^+^ was made by increasing the concentration of KCl to 70 mM. The NaCl concentration was decreased accordingly to maintain osmolality. Imaging was performed using a Polychrome V monochromator (TILL Photonics, Graefeling, Germany) and an Eclipse Ti Microscope (Nikon, Tokyo, Japan) with a ER-BOB-100 trigger on an iXON3 camera and iQ2 (Andor Technology, Belfast, UK) software. Recordings were made at 1 frame/s at 37°C. The cells were perfused during the length of the experiment. Before onset of the experiment an area around each islet was marked and the light intensity was recoded in that area to obtain the integrated light intensity per unit area (in μm^2^) at 340 nm (150 ms exposure) and 380 nm (100 ms exposure). These measured intensities were then used to calculate the ratio of Ca^2+^ bound Fura-2 (340 nm) and unbound Fura-2 (380 nm) at 1 frame/s. The data given is from 16–21 islets from 3 mice per condition (Fig 3).

### RNA extraction and RT-qPCR

**Fig 4:**
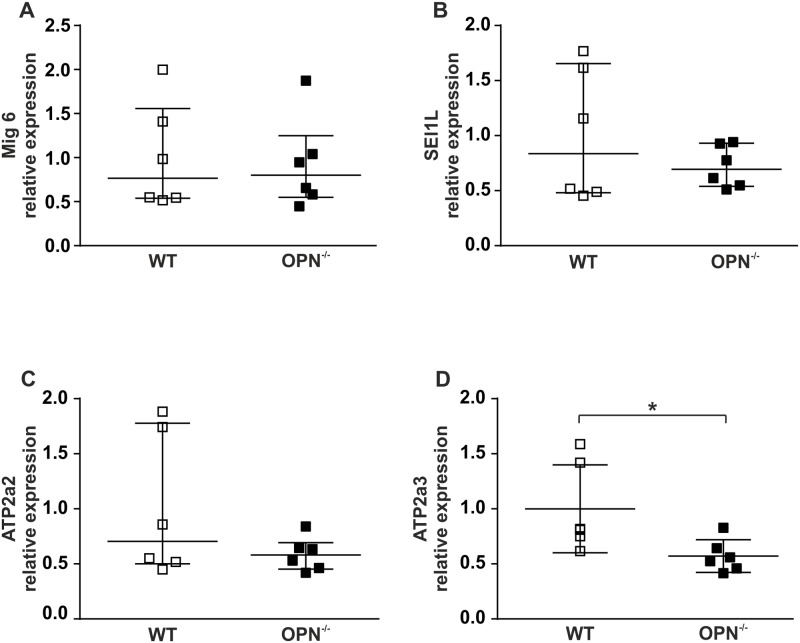
Expression pattern of proteins associated with ER and Ca^2+^ handling in WT and OPN^-/-^ islets. Expression pattern of *Mig 6* (A), *SEl1L* (B), *Atp2A2* (C) and *ATP 2A3* (D) in islets from WT and OPN^-/-^ mice as marked in the figure. Data are described using the median with interquartile range for 6 biological replicates with 3 technical replicates in each experiment. * p≤ 0.05.

Total RNA was extracted from islets with miRNeasy Kits (Qiagen) and quantified using Nanodrop 1000 (Thermo Fisher Scientific). Relative mRNA expression was determined by RT-qPCR. cDNA was prepared using random primers and High Capacity Reverse Transcription Kit (#4368814) and expression was determined using TaqMan Universal PCR Master Mix I with TaqMan gene expression assays (Life Technologies). Amplification and Ct values were obtained using ViiA^™^ 7 Real-Time PCR System and software (Life Technologies). Relative gene expression was normalized against two reference genes. The following TaqMan primers were used: *Atp2a2* Mm01201431_m1, *Atp2a3* Mm00443898_m1, *Sel1l* Mm01326442_m1, *Errfi1* (*Mig 6*) Mm00505292_m1; with reference genes *Ppib* Mm00478295_m1 and *Hprt* Mm00446968_m1. Data are given from 6 mice per condition and each experiment has 3 technical replicates (Fig 4).

### Insulin secretion

**Fig 5:**
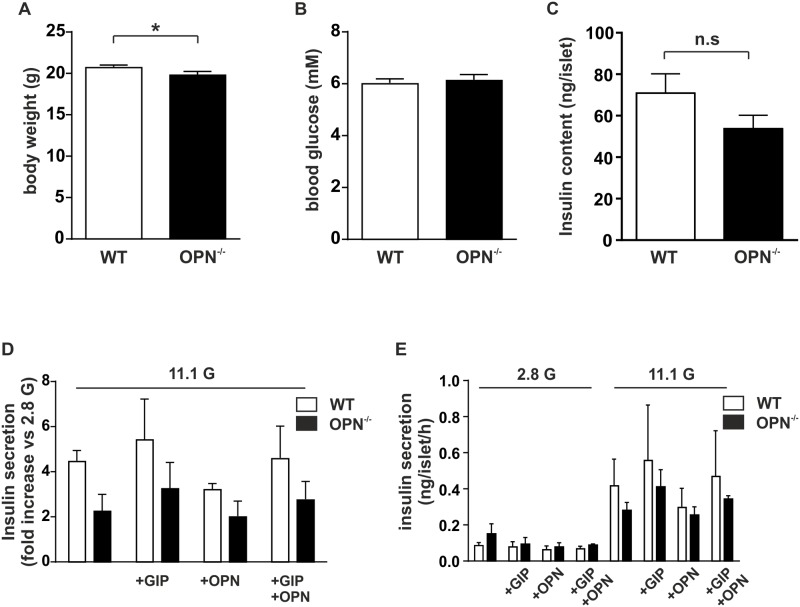
Deletion of OPN has modest effects on metabolism. (A) Body weight in WT and OPN^-/-^ mice measured at 12 weeks. (B) Non-fasted blood glucose levels from WT and OPN^-/-^ mice measured at 12 weeks. (C) Insulin content in isolated islets from WT and OPN^-/-^ mice. (D) Glucose-induced insulin secretion at 11.1 mM glucose from isolated WT and OPN^-/-^ islets described as fold increase over basal insulin secretion at 2.8 mM glucose with and without the addition of 200 ng/ml OPN and/or 100 nM GIP. (E) Same experiment as in (D) but displaying the non-processed data as ng/islet/h. Data are given as mean ± SEM from 28–30 animals (A and B) or from 4 biological experiments with 3 technical replicates in each experiment (C, D, and E). * p≤ 0.05.

Insulin secretion was measured with radioimmunoassay (RIA) using batch incubation of islets essentially as previously described [20]. In short, batches of 10–12 islets were pre-incubated for 30 min in Krebs-Ringer buffer containing 1 mm glucose. This was followed by 1 h incubation in Krebs-Ringer buffer containing 2.8 or 11.1 mm glucose with or without 100 nM gastric inhibitory polypeptide (GIP), 200 ng/ml osteopontin (OPN), or a combination of the two. At the end of the experiment the supernatant was carefully collected and stored at -20°C until further analysis. The batch incubated islets were collected and lyzed in RIPA buffer (150 mM NaCl, 1% TritonX, 0.1% SDS, 50 mM Tris-Cl, 0.5% sodium deoxycholate, 2 mM ADTA and 50nM NaF). The resulting lysate was stored at -20°C until further analysis. Before analysis the lysate was centrifuged at 14000 x g for 15 minutes at 4°C. The resulting supernatant was used to determine insulin content in the samples. Insulin secretion and insulin content were measured using a radioimmunoassay kit (Linco Research Inc MO, USA). Data are derived from 4 biological experiments. Each experiment is conducted on pooled islets from 3–4 animals/condition and with 3 technical replicates in each experiment (Fig 5).

### Data analysis

Data are presented as mean ± SEM except in Fig 4 where they are described as median with interquartile range. Statistical significance was calculated with GraphPad Prism 7 (GraphPad software, CA, USA) using two-tailed Student´s t-test except in Figs 2C, 2D and 4 where we used a Mann-Whitney test and in Fig 5D and 5E where two-way ANOVA using Sidak´s (Fig 5D) or Tukey´s (Fig 5E) multiple comparisons test was used. P values are given as follows: * p≤ 0.05; ** p≤ 0.01; *** p≤ 0.001.

## Results

### Lack of OPN results in reduced number of docked insulin granules

No apparent histological differences were observed between the pancreases of OPN^-/-^ and WT mice, as assessed on hematoxylin and eosin stained sections (Fig 1A). At the level of light microscopy we could confirm that insulin is expressed in the islets of Langerhans in both OPN^-/-^ and WT mice (Fig 1B). Using transmission electron microscopy (TEM) we found that the cells were more loosely connected within the islets from OPN^-/-^ mice compared to WT control. This manifested itself by a higher extent of disrupted islets after preparation for TEM (Fig 2A). A closer examination of the beta cells from the islets lacking osteopontin revealed atypical structures at the plasma membrane of the cells (Fig 2B). These atypical structures would sometimes, but not always, protrude from the plasma membrane. They did not contain insulin containing granules and in their appearance they resembled cortical ER [21], with the difference that in the islets from OPN^-/-^ mice the cortical ER-like structures are rounded rather that longitudinal. Cortical ER is a network of ER close to the plasma membrane. Regions enriched with cortical ER have been suggested to function as hotspots for insertion and cycling of plasma membrane proteins [22] and as regulators of calcium homeostasis [21].

The volume density of the insulin-containing granules (N_V;_ Fig 2C) did not differ between OPN^-/-^ and WT control mice. However, OPN^-/-^ mice had significantly lower number of docked granules (N_S_; Fig 2D). Overall, lack of OPN resulted in a significantly different distribution of granules in relation to the cell membrane, with fewer granules in the vicinity and more granules further away from the cell membrane compared to control (Fig 2E). Distance to the cell membrane was calculated from the center of the granule using an in-house MatLab program.

### Ca^2+^ clearance is altered in islets from OPN^-/-^ mice

The intracellular Ca^2+^ concentrations regulate several aspects of beta cell physiology and it is well established that insulin release is Ca^2+^ dependent. Physiologically, glucose is the most important regulator of beta cell function. We therefore measured intracellular Ca^2+^ fluctuations in islets from OPN^-/-^ mice and WT control during basal and glucose stimulated conditions. As can be seen in Fig 3A and 3B islets from WT and OPN^-/-^ mice display Ca^2+^ oscillations when stimulated with 16.7 mM glucose. We found that cytoplasmic Ca^2+^ concentration under stimulation with 16.7 mM glucose, estimated as area under the graph above baseline, was significantly higher in the OPN^-/-^ mice compared to WT control (333510 ± 42829 AU *vs* 184765 ± 17920 AU; n = 16–21 islets from 3 mice per condition; p ≤ 0.01; Fig 3C). When islets are stimulated with glucose the intracellular Ca^2+^ concentration dips for a short while before the onset of a rapid increase (marked as C_0_ in Fig 3A and 3B). This dip is believed to reflect uptake of Ca^2+^ into the ER [23]. We measured the area of the dip below baseline and found that it was significantly smaller in islets from the OPN^-/-^ mice compared to control (3072 ± 483 AU *vs*. 8863 ± 983 AU; n = 16–21 islets from 3 mice per condition; p ≤ 0.001; Fig 3D). We saw no significant difference in the amplitude of the first peak (marked as C_1_ in Fig 3) between OPN^-/-^ and WT islets (0.41 ± 0.03 AU *vs*. 0.33 ± 0.03 AU; n = 16–21 islets from 3 mice per condition; Fig 3E) or the frequency of oscillations during the second phase (1.9 ± 0.3 *vs*. 2.5 ± 0.2 peaks/minute; n = 16–21 islets from 3 mice per condition; Fig 3F). Although the frequency of oscillations did not differ between the different islet types, the amplitude of these oscillations did. In islets from the WT mice 14 out of 16 islets displayed oscillations with an amplitude > 0.1 AU while the same number for islets from the OPN^-/-^ mice is 7 out of 21. Importantly, all islets displayed oscillations in these experiments.

### The expression of the SERCA3 encoding gene *Atp2a3* is reduced in OPN^-/-^ islets

The ultrastructural data and the Ca^2+^ imaging data point towards ER alterations in islets from the OPN^-/-^ mice. We therefore performed qPCR on isolated islets from these mice to study the expression of selected genes related to ER function and ER stress. We focused on the genes *Mig 6*, *Sel1L*, *Atp2A2*, and *Atp2a3*. *Mig 6* encodes for mitogen-inducible gene 6 protein. This protein is believed to play a role in the cellular stress response and is upregulated in pancreatic beta cells during ER stress [24]. *Sel1L* encodes for a protein that takes part in ER associated degradation of misfolded proteins. Moreover, in ER from cells lacking *Sel1L* the structures are more rounded much like the atypical structures in Fig 1B [25]. *Atp2A2*, and *Atp2a3* both encode for SERCA Ca^2+^ ATPases, which are Ca^2+^ pumps situated in the ER membrane [26]. As can be seen in Fig 4, expression of *Atp2a3* was significantly lower in OPN^-/-^ islets compared to WT whereas the other three genes tested did not show significantly altered expression levels. It is interesting to note that *Sel1L*, *Atp2A2*, *and Atp2a3* all exhibited a significantly lower variance in their expression levels in the OPN^-/-^ mice compared to WT control (p ≤0.05 for *Sel1L* and *Atp2a3*, and p ≤0.01 for *Atp2A2*).

### Insulin secretion is not altered in islets from OPN^-/-^ mice compared to WT islets

Finally, we examined whether depletion of OPN resulted in a metabolic phenotype. At 12 ± 1 week we measured body weight and blood glucose in the OPN^-/-^ mice. There was a slight but significant decrease in body weight (Fig 5A) but no difference in blood glucose (Fig 5B) in the OPN^-/-^ mice compared to control.

We next isolated islets from the mice and examined these for their ability to produce and secrete insulin. No significant difference in insulin content in OPN^-/-^ islets compared to WT control was observed (Fig 5C). When evaluating the fold increase of insulin secretion compared to 2.8 G we found that although there is a tendency to reduced insulin secretion in the OPN^-/-^ islets this did not reach significance. Neither islets from OPN^-/-^ mice nor islets from WT control responded to acute addition of OPN and/or GIP (Fig 5D).

There was a tendency to an increased basal insulin secretion in the OPN^-/-^ islets compared to WT control and therefore insulin secretion expressed as (ng/islet/h) for 2.8 and 11.1 mM glucose with and without addition of OPN and GIP is displayed in Fig 5E.

## Discussion

Osteopontin is a versatile protein found in many tissues throughout the body. Extracellular OPN exerts its effects by binding to cell surface receptors. Intracellular OPN is less well studied and if and how it affects cell physiology is largely unknown. Mouse pancreatic beta cells express OPN [7]. The pancreatic beta cells have been assigned the important task of producing and secreting insulin. Insulin is the major blood glucose lowering hormone in the body and as such its production and release is under tight regulation by a multitude of mechanisms. In this study we asked what role OPN plays in beta cell physiology with a special focus on insulin secretion.

Although islets from OPN^-/-^ mice at a first glance appear normal, the OPN^-/-^ islet cells have weakened cell-cell connections. This is probably due to the fact that OPN is an extracellular matrix protein that normally binds integrins [27]. It therefore seems plausible that a loss of OPN destabilizes the islet structure. Within the islet beta cells are connected through gap junctions [28, 29]. It is widely recognized that these gap junctions play a role in the synchronization of beta cells within the islet and accordingly the connexin36 knock-out mice, which lacks gap junctions, do not display Ca^2+^ oscillations upon stimulation with glucose [30]. Interestingly, islets from OPN^-/-^ mice display synchronized Ca^2+^ oscillations. Hence, the weaker islet-islet connections in the OPN^-/-^ mice do not seem to affect beta cell communication. We therefore suggest that the physiological effect of this phenomenon is minor during normal conditions. It would be interesting to investigate if the weaker cell-cell connections give a more prominent physiological effect in OPN^-/-^ islets subjected to stress, for instance the low grade inflammation suggested to occur in T2D.

We show that Ca^2+^ handling differs between beta cells in OPN^-/-^ and WT islets. In response to 16.7 mM glucose, initial Ca^2+^ clearance into ER was diminished and intracellular Ca^2+^ concentration was increased in OPN^-/-^ islets compared to WT control. Beta cells contain a well-developed ER that takes up Ca^2+^ through SERCA 2A and SERCA 3. The ER is then known to release the Ca^2+^ again either as a response to specific signals or through an undefined leaking process [23]. As a crude measurement for ER function we determined the expression of ER related genes encoding for the two SERCAs (*Atp2A2*, and *Atp2a3*) as well as genes involved in ER stress and clearance of misfolded proteins (*Mig6* and *Sel1L*). Islets from OPN^-/-^ mice displayed a lower expression level of *Atp2a3* which is in line with the increased glucose-stimulated cytoplasmic Ca^2+^ concentration in these islets. This speaks in favor of the hypothesis that the reduced Ca^2+^ clearance is due to reduced influx of Ca^2+^ to the ER rather than to an increased Ca^2+^ leak from the ER back into the cytoplasm. In the SERCA 3^-/-^ mouse the initial dip generated by elevated glucose was not altered [31]. Therefore the reduction in *Atp2a3*, which encodes for SERCA 3, does not explain the reduced initial dip recorded in the OPN^-/-^ islets. The SERCA 3^-/-^ mouse shows increased glucose-stimulated insulin release [31]. We do not detect altered insulin secretion from the OPN^-/-^ islets in our measurements. This could be because the reduction in SERCA 3 is too small to generate an effect on insulin secretion. Another explanation could be that beta cells from OPN^-/-^ mice display fewer docked granules than WT control and in general fewer granules near the plasma membrane. Before insulin can be released through exocytosis, insulin containing granules need to translocate to the plasma membrane where they dock before they release their cargo. There are several examples in the literature where reduced translocation and docking of granules lead to reduced insulin secretion [32, 33]. It is possible that the OPN^-/-^ beta cells can compensate for the reduced amount of docked granules under normal conditions. But it could also be that any negative effects on insulin secretion due to the reduced amount of docked granules is neutralized by increased intracellular Ca^2+^ and reduced expression of *Atp2a3*.

Near the plasma membrane the beta cells from OPN^-/-^ mice showed atypical morphological changes. We believe that these morphological changes are the reason why OPN^-/-^ mice have fewer granules close to or at the plasma membrane as they would act as physical barriers for the insulin vesicles. Accordingly, no docked vesicles were observed at these morphologically changed sites. It has been reported that ER Ca^2+^ depletion in HeLa cells resulted in a ~4 fold increase of ER close to the plasma membrane (cortical ER) [21]. In analogy with this, if the islets from the OPN^-/-^ mice have reduced ER Ca^2+^ because of reduced Ca^2+^ uptake (reduced initial C_0_; Fig 3) this might then have triggered translocation of ER to the cell membrane. One effect of such ER translocation would probably be an increase in store-operated calcium entry (SOCE) [34]. Islets from OPN^-/-^ mice have increased intracellular Ca^2+^. This is probably due to reduced uptake of Ca^2+^ into the ER possibly in combination with increased SOCE. Long term increase in intracellular Ca^2+^ can trigger apoptosis [35]. It has been reported that OPN have anti-apoptotic effects through the inhibition of NO production [4, 6]. In light of our findings it is possible that a role for OPN in the regulation of intracellular Ca^2+^ adds to this effect.

The variation in gene expression between individuals was markedly reduced in *Sel1L*, *Atp2A2*, and *Atp2a3* in islets from the OPN^-/-^ mice compared to WT control. General differences in gene expression levels between knock-out models and their WT controls has been described in the literature [36]. It is not possible in the present study to determine if the variation in gene expression is specifically due to the removal of OPN or a general effect because we are using knock-out animals.

We have previously shown that gastric inhibitory polypeptide (GIP) increases the expression of OPN in mouse beta cells [7]. A similar relationship between GIP and OPN is also present in other tissues [37]. In our study we could not find any evidence that short term (1 h) stimulation with GIP affected glucose-induced insulin secretion differently in OPN^-/-^ islets compared to control. It has previously been reported that addition of OPN to islets from mildly diabetic mice [4] or to cytokine treated rat islets [6] restores glucose-induced insulin secretion. At first sight this might seem contradictory to our results, but in those studies the islets were incubated in OPN overnight while we acutely added OPN during the 1 h glucose-stimulation of the islets. Therefore, our experiments only investigate immediate regulation of insulin release while processes that need longer to occur are left unexplored.

OPN^-/-^ mice on high fat diet have been reported to gain less weight [38] and develop less insulin resistance [39] compared to controls. In our study the mice were kept on a normal diet and yet the OPN^-/-^ mice displayed a small but significant reduction in body weight compared to WT control. This corroborates and expands what is previously published [38] and again highlights the possibility of using OPN as a molecular target to control body weight. We did not investigate insulin sensitivity in the OPN^-/-^ mice, but the fact that blood glucose was normal in combination with, if anything, a slight insulin secretory defect indicates that we have no changes or possibly a slight improvement in insulin sensitivity in the OPN^-/-^ mice.

In summary, our study shows that removal of OPN from mouse pancreatic beta cells results in negative alterations of beta-cell physiology. Under normal, healthy circumstances these negative alterations appear to be compensated for. However, the elevated Ca^2+^ levels detected in the OPN^-/-^ mice with the possible implications of a compromised ER combined with the altered insulin vesicle localization indicates that reduced levels of OPN could in the long run be a factor contributing to development of beta cell failure. Moreover, we and others have previously shown that OPN has an islet and beta cell protective role [4, 6, 7]. Therefore, while OPN has been suggested as a target to reduce insulin resistance and other obesity related disorders [40] the potential risk of beta cell defects should be considered.
